# Characterizing the Structural Evolution of Cereal Trade Networks in the Belt and Road Regions: A Network Analysis Approach

**DOI:** 10.3390/foods11101468

**Published:** 2022-05-18

**Authors:** Wei Chen, Haipeng Zhang

**Affiliations:** 1 Institute of Geographic Sciences and Natural Resources Research, Chinese Academy of Sciences, Beijing 100101, China; chenw@igsnrr.ac.cn; 2 Key Laboratory of Regional Sustainable Development Modeling, Chinese Academy of Sciences, Beijing 100101, China; 3 College of Resource and Environment, University of Chinese Academy of Sciences, Beijing 100049, China

**Keywords:** food security, international food supply chains, cereal trade network, cereal industry, cereal trade market, the Belt and Road

## Abstract

Cereal trade is essential for economic and commercial cooperation among countries along the “Belt and Road” (BRI). It helps ensure food security and contributes to building a community of interests and destinies for the BRI countries. Based on the UN Comtrade database, this study, using a network analysis approach, investigates the structural characteristics and spatiotemporal dynamics of cereal trade networks among the “Belt and Road” countries. Results show that: (1) The cereal trade among the BRI countries has formed well-connected and complex trade networks, and the “Belt and Road” initiative has significantly promoted cereal trade networks among the BRI countries. (2) The backbone structures of cereal trade networks along the BRI are in geographical proximity. India, Russia, and Ukraine are the most important trading partners and absolute core nodes in the trade networks, influencing the entire cereal trade networks. (3) The BRI cereal trade networks exhibit significant core-periphery structures, with considerable power asymmetries between the countries reflecting food supply and demand differences. In general, the BRI cereal trade networks have developed from relatively diversified to polarized. Supply chains in the cereal trade network are dominated by a few large countries and are fragile, with weak resilience and low resistance to risk. Therefore, governments should continue to strengthen regional cooperation, optimize cereal trade network structure, enhance their reserve capacity, and build a stronger system to guarantee food security and prevent risk. All these measures will support the food security of the “Belt and Road” countries.

## 1. Introduction

Food is fundamental to human survival and development, and its abundance—or lack thereof—directly affects a country’s social stability and economic development. Therefore, topics related to food security, such as food production, distribution, trade, and storage, have long been a central concern of government departments and academics worldwide. Food production is influenced by soil and water resources, climatic conditions, and production technology, and has deep regional roots [1,2,3]. Globally, the distribution of food production and per capita food production in different countries is highly uneven. However, the spread of economic globalization and the free trade of cereal have led to a worldwide redistribution of the agricultural resources on which cereal production depends, providing a meaningful way to regulate the geographical imbalance between food supply and demand [4,5,6,7,8]. The volume of cereal traded internationally has more than doubled over the past three decades, with a rising number of countries now importing or exporting cereal [9,10]. As a result, the food security status of a particular country or region is interlinked with many others. Therefore, maintaining the stability of the international cereal trade network has an important role in promoting the security and stability of countries around the world and building a community of human destiny.

Social network analyses can quantitatively assess complex linkages in socio-economic development [11]. They align with research approaches that explore changes in the patterns and processes of geographical phenomena. Recently, network analysis tools have been used widely to explore the increasing connectivity of global food trade networks [12,13,14], with the study of agricultural trade networks as the most common research object. For instance, Cai and Song applied a complex network to analyze the relationship between agricultural and national trade and found that agricultural trade facilitates international commerce [15]. Wang et al. studied 57 agricultural products from the six categories of cereal, oilseeds, fiber, sugar, fruits and vegetables, and meat. They noted the rising density of global agricultural trade networks and their increasing diversification [16]. Shutters and Muneepeerakul applied social network analysis to investigate international agricultural trade networks and their relationship to patterns of international development [17]. As research continues, further trade network studies of various food types have emerged, including those focusing on seafood [18], meat [19,20], cereal [21], and soybean [22]. Research into the growing segmentation of trade networks involving wheat [23,24], maize [25], and rice [26] provides important theoretical support for developing more beneficial, evidence-based food security strategies. In terms of research methods, many studies have used quantitative measures of network characteristics, such as node strength, network density, betweenness centrality [27,28,29], community detection [30,31], and core-periphery structure [32,33]. In addition to these fixed research methods, researchers have applied invariant subnetwork structures [34], complex network research frameworks that combine trade efficiency and elasticity trade-offs, and other combinatorial research methods [35] that tap into specific network structures.

The “Belt and Road” initiative (BRI) aims to build a new platform for international cooperation and exchange that is open, inclusive, equal, and mutually beneficial [36,37]. After nearly 10 years of promotion and construction, the BRI has achieved remarkable results in promoting economic and trade exchanges and open cooperation among countries along the route [38,39]. As an important element of these ties, the cereal trade along the BRI has also captured the attention of scholars. Successive studies have investigated the utilization of cereal markets and resources in the BRI region [40], cereal trade and its implied virtual water and soil resources [41], and spatiotemporal patterns of cereal production and consumption [42]. The agricultural development of BRI countries is characterized by limited use of technology, land-use efficiency, and production organization. Some of these countries struggle to feed their populations as a result, strengthening the demand for trade in cereal between them [43,44]. Therefore, further research on transnational agricultural cooperation and food trade networks is important to meet the BRI countries’ objective needs and to provide insights on integration points for countries along the route to building a community of interests and destiny together.

Existing research has made various advances that enrich and expand the knowledge of international cereal trade networks. However, a synthesis of the available studies exposes several deficiencies. First, compared with research output on BRI trade networks in general, studies focusing on cereal are relatively scarce, and relevant knowledge is limited. Earlier studies of cereal trade networks explored soil and water resources that are implied by the pattern of trade. Moreover, they analyzed spatiotemporal food production and consumption patterns, while network studies of structural changes in regional cereal trade along the BRI have yet to be supplemented. Second, while the characteristic pattern and topological relationships of cereal trade networks have been examined, the unevenness of cereal trade network structures and the large differences in trade volume and linkages between different countries have been neglected. Furthermore, few studies have employed methods that reveal the inner structural characteristics of cereal trade networks and their evolutionary processes. An in-depth understanding of the structure of the cereal trade network will provide important practical guidance for enhancing and expanding trade and agricultural cooperation among the BRI countries. Finally, despite the importance of the cereal trade to preserving regional food security, studies emphasized the portrayal of patterns rather than trade network issues and risk assessment. This research was theoretical rather than practical, limiting its ability to guide users faced with the dynamics and instabilities of the food security situation.

To fill this gap, this paper describes an approach that integrates a top network method, centrality analysis, and core-periphery profile to portray the patterns of cereal trade networks among countries along the BRI since 2001. It analyzes the main structures of the cereal trade networks between these countries, identifies the evolution of their core-periphery structures, and details their structural evolution. The research findings are expected to enhance scientific understanding of the structural evolutionary characteristics of the BRI cereal trade network and inform cereal trade cooperation, building a community of cereal production and consumption, and ensuring the food security of countries along the BRI.

## 2. Materials and Methods

### 2.1. Study Area

The BRI is committed to building an open and inclusive platform for cooperation and is not limited to specific regions [38,39,45]. However, according to the actual research needs and with reference to the geographical scope of previous related studies, the BRI countries in this paper refer to the traditional 65 countries along the BRI. Table 1 lists the specific countries and regions.

### 2.2. Analytical Framework

The BRI plays an essential and positive role in promoting regional trade integration, while agricultural cooperation, especially food trade, has been a critical element of trade cooperation among countries along the Silk Road since ancient times. This paper employed top network, centrality, and core-periphery profile algorithms to quantitatively evaluate the structural characteristics and spatiotemporal dynamics of cereal trade networks in countries along the BRI. First, we constructed a trade network matrix of 65 countries along the BRI, visualized the spatial network of cereal trade in countries along the BRI, and revealed the overall pattern evolution of the cereal trade network from a geographical perspective. Second, we extracted the top network of the BRI cereal trade network to capture the backbone trade structure constituted among the largest trading countries and visualized the representation using Gephi software. Moreover, we measured the trend of centrality evolution of BRI cereal trade top network nodes by applying characteristic metrics in network science, such as degree centrality, betweenness centrality, and eigenvector centrality. Finally, we use the core-periphery profile algorithm to analyze the core and peripheral structures of the full cereal trade networks and identify the most influential and peripheral countries and regions in the BRI cereal trade network. Based on the above-described analytical framework, we comprehensively explore the spatiotemporal dynamics of the cereal trade network structure along the BRI.

### 2.3. Data Processing

As the primary food source for humans, cereal plays a fundamental role in feeding populations and accounts for the largest share of all agricultural trade. Therefore, in this paper, we adopt the narrow sense of food, as mentioned in this paper, which refers to cereal. Consequently, we select “cereal” (HS10) as specified in the International Convention for Harmonized Commodity Description and Coding System, which includes wheat and meslin (HS1001); rye (HS1002); barley (HS1003); oats (HS1004); maize (HS1005); rice (HS1006); grain sorghum (HS1007); and buckwheat, millet, and canary seed (HS1008). The trade flow data were derived from the “cereal” data in the UN Comtrade database. We considered countries as nodes in the network and trade flows between countries as edges. First, we constructed a 65 × 65 trade network matrix based on the spatial scope of the BRI. Second, we matched the trade flows between countries to the network matrices and constructed directed and weighted trade network matrices. Finally, we transformed the directed and weighted matrices into undirected and weighted matrices, and then constructed the BRI cereal trade network dataset since 2001.

Considering year-on-year variation in international cereal trade, this paper selects and compares four representative years highlighting how the BRI cereal trade networks have evolved in different contexts. China’s accession to the World Trade Organization in 2001 dramatically altered global trade patterns and was, therefore, the starting point of the study. Furthermore, the 2008 global financial crisis and China’s proposal of the BRI in 2013 profoundly affected the global economy and the division of labor, significantly impacting the cereal trade network across these countries. Finally, the COVID-19 pandemic that began at the end of 2019 exerted a considerable impact on the world economy and global trade [46]. Therefore, 2019 was considered as the end year of this study. The selection of these four representative years is, on the one hand, conducive to revealing the key characteristics of the international cereal trade system prior to the COVID-19 pandemic and summarizing general patterns, and on the other hand, guarantees the timeliness of the study and provides a comparison and reference for subsequent studies on the impact of global crisis events on trade networks. For these reasons, we focused on 2001, 2008, 2013, and 2019 as the representative years for the study.

### 2.4. Methods

#### 2.4.1. Top Network

The top network is a simple and effective method for extracting the backbone structure in a network. It is a subnetwork that consists of the strongest connections of each node from the complete network, which can reduce the masking of many weak connections within the network to the overall information of the network structure [38,47]. Top1 network refers to the fact that only trade links between countries with the highest trade volume in each country are retained in the entire trade network. Top2 network refers to the trade links between only the top two countries in terms of trade volume in each country in the entire trade network. Although the construction of the top network omits certain trade connections, it retains the backbone connections of the whole trade network and achieves the feature of portraying most of the global trade network structure with fewer trade connections. Based on the analysis of the structural characteristics of the cereal trade network, we selected the top network to characterize the evolution of the cereal trade network of the BRI countries in the years selected for this study.

#### 2.4.2. Network Centrality

(1) Degree Centrality

Degree centrality refers to the number of nodes directly connected to a particular node in a network. As an indicator, it portrays the strength of a node’s connection to other nodes [21]. In general, the greater the degree centrality, the more connections are established with that node in the network and the greater the importance of that node within the network.

(2) Betweenness Centrality

Betweenness centrality measures the importance of nodes as intermediaries in a trade network. The higher the betweenness centrality, the stronger a node’s role as a bridge in a network and the greater its potential ability to control other nodes. In a network with *N* nodes, the shortest path between nodes *j* and *k* will access certain nodes; if node *i* is passed by many shortest paths, it indicates that the node is important in the network. The importance can be expressed in terms of the betweenness centrality *B.C. (i)*, which is calculated as [27]:

(1) $$C_{B}\left(i\right)=\overset{}{\underset{\begin{array}{c} 1\leq j\leq k\leq N\\ s\neq j\neq k \end{array}}{\sum }}\frac{n_{jk}\left(i\right)}{n_{jk}}$$

where $n_{jk}$ is the number of shortest paths between nodes *j* and *k*, and $n_{jk}\left(i\right)$ is the number of nodes *i* through which the shortest path between nodes *j* and *k* passes.

(3) Eigenvector Centrality

The importance of a node in a network depends not only on its centrality, but also on the number and centralities of its neighboring nodes. Eigenvector centrality measures the centrality of a destination node in terms of the centrality of the connected nodes around the destination node. It is an indicator of the connectivity of the nodes in a network. A node with a high eigenvector is connected to many nodes that themselves have high eigenvectors. With reference to Ge et al.’s [48] study, eigenvector centrality is defined as:

(2) AX=ZX

(3) $$Z_{i}x_{i}=a_{1i}x_{1}+a_{2i}x_{2}+\cdots +a_{it}x_{i}+\cdots +a_{ni}x_{n},\;(i\neq t)\;$$

(4) $$C_{\left(e\right)i}=Z_{i}$$

where *A* is an *n* × *n* adjacency matrix composed of *a_ij_*, *X* = (*x*_1_, *x*_2_, *x*_3_, …, *x*_*n*_)^*T*^ denotes the degree centrality of each node, respectively, and *Z_i_* is the eigenvector centrality value, while *a_ij_* denotes the contribution of node *i* to the status of node *j*. *C_(e)i_* denotes the eigenvector centrality of node *i*.

#### 2.4.3. Core-Periphery Profile

The portrait of a network as divided into a dense core and a sparse periphery, referred to as a core-periphery structure, originated from scholars in social sciences in the 1990s, and the paradigm has since been extended to other disciplines [49]. To identify the core-periphery structures in networks, various algorithms have been successively proposed, including block-modelling [50], k-shell decomposition [51], and centrality [52]. However, most of the proposed algorithms are incapable of dealing with weighted networks, and their robustness still needs to be verified. Against this background, Della Rossa et al. recently proposed the algorithm of core-periphery profile [53], disclosing the overall network structures and the peculiar roles of specific nodes.

In a network with an ideal core-periphery structure, peripheral nodes (p-nodes) are allowed to link to core nodes only. In other words, no connectivity exists among p-nodes. However, in most real-world networks, the structure is not ideal, although the core-periphery structure is evident: A weak (but not null) connectivity exists among the peripheral nodes. This calls for the generalized definition of α-periphery, which denotes the largest subnetwork S with the persistence probability $\alpha_{S}\leq \alpha$ .

We define the core-periphery profile *α_k_*, *k = 1*, *2*, *…*, *n*, of the network using the following algorithm [13]:

(5) $$\alpha_{k}=\underset{h\in N\backslash P_{k-1}}{\min}\frac{\sum_{i,j\in P_{k-1}\cup \left\{h\right\}}\pi_{i}m_{ij}}{\sum_{i\in P_{k-1\cup \left\{h\right\}}}\pi_{i}}\;=\underset{h\in N\backslash P_{k-1}}{\min}\frac{\sum_{i,j\in P_{k-1}}\pi_{i}m_{ij}+\sum_{i\in P_{k-1}}\left(\pi_{i}m_{ih}+\pi_{h}m_{hi}\right)}{\sum_{i\in P_{k-1}}\pi_{i}+\pi_{h}}$$

We start with the node *i* with the weakest connectivity, and generate a sequence of sets *{1} = P1* ⊂ *P2* ⊂ *…* ⊂ *Pn = N* by adding, at each step, the node that attains the minimal increase in the persistence probability. Correspondingly, we obtain the core-periphery profile, that is the sequence $0=\alpha_{1}\leq \alpha_{2}\leq \cdots \leq \alpha_{n}=1$ of the persistence probabilities of the sets *P_k_*.

The above algorithm provides, as byproducts, two other important tools of analysis, including centralization and coreness. We define the centralization *C* for a core-periphery profile $\alpha_{k}$ as the complement to 1 of the normalized area, namely [13]:

(6) $$C=1-\frac{2}{n-2}\overset{n-1}{\underset{k=1}{\sum }}\alpha_{k}$$

Therefore, we can quantify this similarity by measuring the area between the $\alpha_{k}$ -curve of a given network and of the star network and normalizing to assign *C = 1* to the star network itself (maximal centralization) and *C = 0* to the complete network (no centralization). If a network displays a definite core-periphery structure (large *C*), then the sequence $\alpha_{k}$ naturally provides a measure of coreness of each node. We have $\alpha_{k}$ = *0* for all p-nodes (the periphery in the strict sense), whereas the coreness of the last inserted node is maximal and equal to $\alpha_{n}=1$ .

## 3. Results

### 3.1. Cereal Trade Patterns of the BRI Region

**Figure 1:**
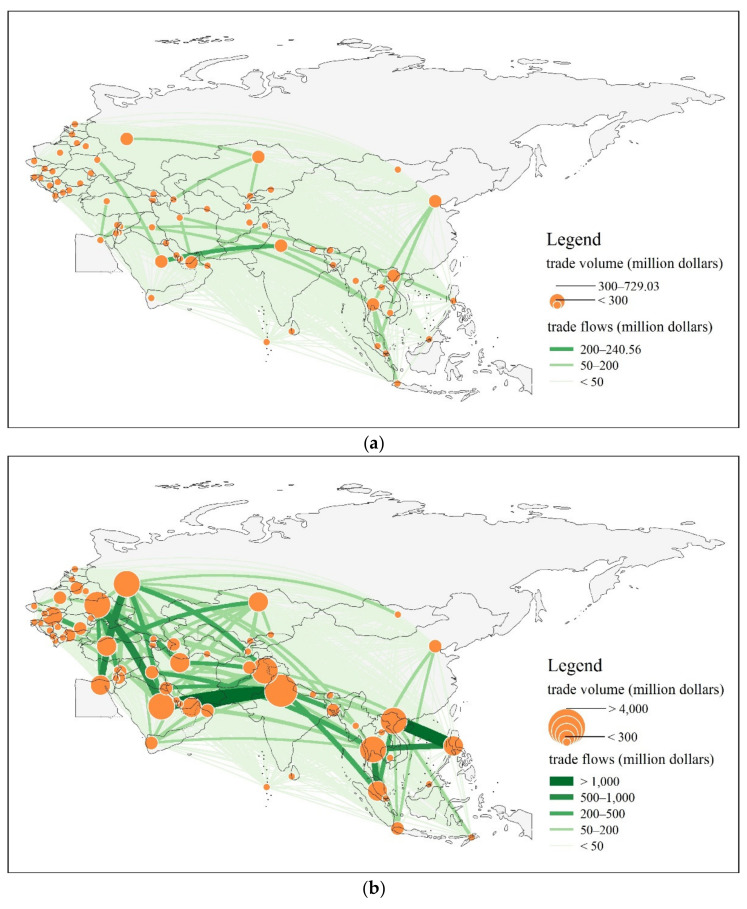

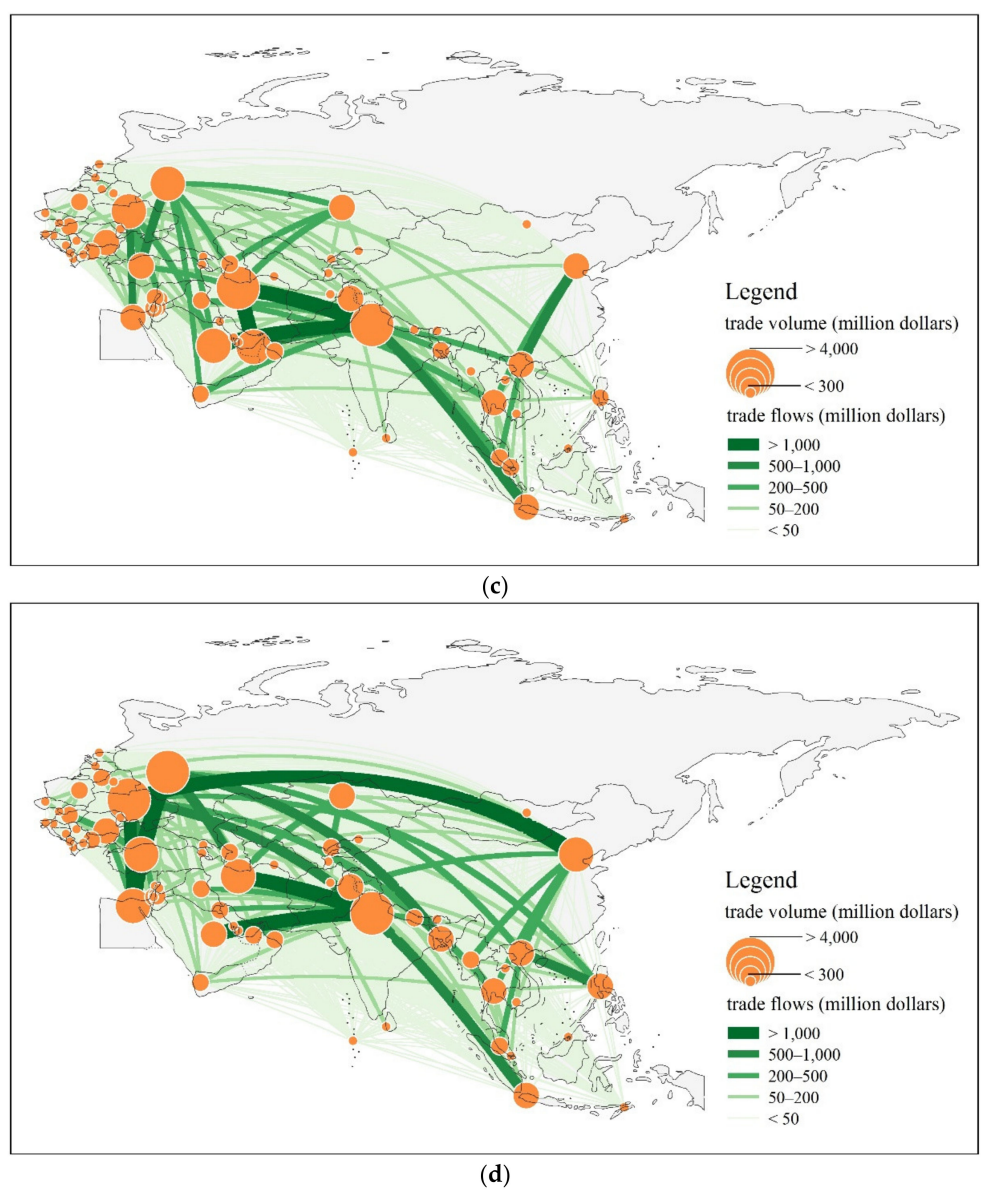
The cereal trade networks in the BRI region (**a**) in 2001, (**b**) in 2008, (**c**) in 2013, and (**d**) in 2019. Data source: https://comtrade.un.org/data/ (accessed on 14 December 2021), illustrated by the authors.

Considering countries as nodes and cereal trade flows of each country to other countries as edges, we constructed complete undirected and weighted cereal trade networks among 65 countries along the BRI during 2001–2019. Network weights refer to the trade flows between countries, reflecting the scale of trade between pairs of countries; the thickness of the edge lines represents weights. The cereal trade networks between countries along the BRI are visualized in Figure 1. Overall, the cereal trade flows among countries along the BRI formed dense and complex trade networks over the period, and the BRI significantly promoted these linkages from 2013.

The cereal trade in the BRI region continued to grow in value, from $3.809 billion in 2001 to $28.51 billion in 2019. The most remarkable growth occurred between 2001 and 2008, when trade increased nearly four-fold. More moderate growth of only $1.028 billion was recorded between 2008 and 2013. However, after 2013, the growth of the cereal trade in the BRI region accelerated, increasing by $5.084 billion in 6 years, which was almost five times the increase recorded between 2008 and 2013. Therefore, the density and complexity of the cereal trade network across the BRI countries grew enormously between 2001 and 2019, reflecting the vastly increased volume and flow of trade in these commodities. Since 2013, in particular, the interdependence of cereal trade between the countries deepened, backbone connections grew, and network structures developed significantly. To further reveal the scale characteristics of cereal trade along the BRI, we now explore two aspects in detail: Total trade volume, which refers to the total import and export of cereals within one country, and trade flow, which denotes the total trade of cereals between two countries where cereal trading occurs.

The trade value of the BRI countries has significantly increased, and core nodes have great influence. From 2001 to 2019, the cereal trade of the first node grew in value from $729 million to $6683 million, while the number of nodes with total trade in excess of $100 million in 65 countries grew from 23 to 52. India, Russia, and Ukraine are the core nodes of cereal trade along the BRI, and the scale of their trade has long been among the top countries along the route. Moreover, the clustering characteristics of cereal trade in countries along the BRI are evident, and the primacy of the network system is increasing. The trade value share of the top node fluctuated from 19.14% in 2001 to 23.44% in 2019, and the cumulative share of the trade value of the top five nodes has continued to grow from 69.31% to 87.73%, with significant polarization characteristics of cereal trade.

There has been a strengthening of the trade connections between countries along the BRI, leading to a significant increase in trade flow. In 2001, the largest trade link was the cereal trade between India and Saudi Arabia, with $241 million. Cereal trade flows in the BRI countries have increased in line with the deepening of economic globalization and the increasing openness of countries. Indeed, by 2019, the largest cereal trade link between countries was established between Turkey and Russia, with a trade value of $1.658 billion. Furthermore, the backbone trade linkages have evolved from a single structure to a diversified one, increasing the cereal trade interdependence between countries. The largest trade connection in 2001 had a value of less than $300 million; this was also significantly greater than the trade links between other countries along the route. However, by 2008, there were three connections with more than $1 billion in trade. In 2013, the number of connections with more than $1 billion in trade remained the same; the size of the trade connections at different levels had increased. Since the BRI was proposed, the intensity of the BRI cereal trade links has increased significantly; six trade links have a flow of more than $1 billion, namely Turkey and Russia, Russia and Egypt, Iran and India, Ukraine and Egypt, Saudi Arabia and India, and Ukraine and China, with a significant trend toward the diversification of backbone links.

### 3.2. Structural Evolution of the Top Networks

**Figure 2:**
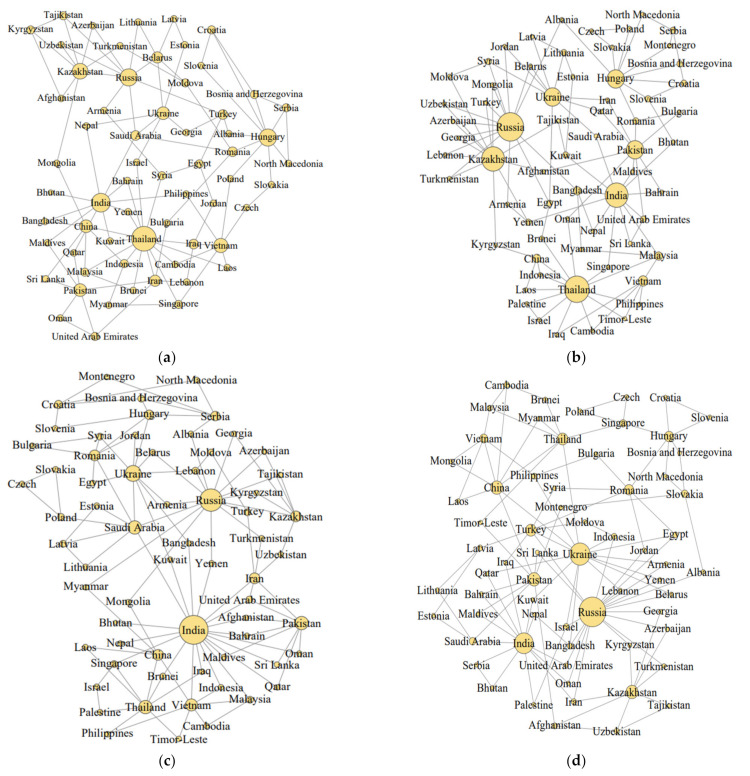
The top network structures of cereal trade networks in the BRI region (**a**) in 2001, (**b**) in 2008, (**c**) in 2013, and (**d**) in 2019. Data source: https://comtrade.un.org/data/ (accessed on 14 December 2021), illustrated by the authors.

Based on the cereal trade networks, we calculate the top1 and top2 networks from the original trade network, respectively. With the change of years, the top1 and top2 networks only account for 1.5% and 3.1% of all network links, but the trade values account for 50% and 70%, respectively. Therefore, we select the top2 network to analyze the main features of the whole network. Furthermore, the top2 networks are then visualized using Gephi software to portray the structural evolution of the backbone structures formed among the largest trading partners. In the backbone structure (Figure 2), nodes denote individual countries, and edges denote trade links between countries. The size of the nodes is proportional to the number of external trade relations of the countries; the larger the nodes, the more external links the country has in the backbone network.

First, the results show that the backbone structures of the cereal trade networks in the BRI region were characterized by geographical proximity throughout the period. The five Central Asian countries, Mongolia, and Russia have formed regional trade blocs with Kazakhstan and Russia as trade centers. Similar regional blocs were formed by the Central and Eastern European countries, with Hungary and Ukraine as centers. Due to its weight and volume, cereal is a freight-sensitive commodity, and geographical distance continues to play a vital role in regional trade interactions. Second, India, Russia, and Ukraine were the most crucial cereal trading partners among the BRI countries, and as time progresses, Russia’s trade position is becoming increasingly important. Over the 18-year period, the above three countries have become the top two trading partners of nearly 10 countries, and their number of partner countries in the top2 trade network has been growing. Russia’s partner countries in the top2 trade network have grown from 9 in 2001 to 19 in 2019, topping all countries. In addition, the main trading partners of some countries changed throughout the period, indicating the overall dynamism and competitiveness of the cereal trade market along the BRI. As Figure 2 shows, the core trading countries evolved from relatively pluralistic to polarized, except for some core nodes that held leadership positions for a long time. However, countries, such as Thailand and Hungary, have gradually lost their original influence within the trade networks.

### 3.3. Centrality Characteristics of the Trade Networks

In this section, we identify the hierarchical structures of the top2 networks of the BRI cereal trade with the help of three indicators: Degree centrality, betweenness centrality, and eigenvector centrality, along with the specific attributes of the network structures that each indicator can tap.

Table 2 shows the top 10 countries and their values for each centrality indicator in 2001, 2008, 2013, and 2019. The most significant centrality characteristics of individual countries are now described. First, Russia, India, and Ukraine were the absolute centers of the cereal trade networks in the BRI region. These three countries consistently recorded the highest values for the three types of centrality indicators, and thus controlled the entire trade network structures. Second, China and Kazakhstan were also important to the BRI cereal trade networks due to their high and stable degree and betweenness centrality indexes. They exerted significant influence across the entire network and played an important bridging role in the cereal trade between countries. Third, two countries in Central and Eastern Europe—Romania and Hungary—assumed the important role of intermediaries or gatekeepers. Although their degree centrality and influence within the whole trade network were limited, their betweenness centrality ranked among the highest of all countries along the route, strengthening these countries’ potential for controlling the cereal trade of other nodes. Finally, Pakistan gained a more prominent influence within the entire trade network, while its degree centrality was among the highest of the BRI countries. However, the dynamics of Pakistan’s betweenness centrality changed frequently, and it played a more restricted role in the trade network.

Over time, the positions of some countries in the cereal trade networks along the BRI shifted significantly. First, the influence of Southeast Asian countries gradually decreased, with Thailand’s degree centrality decreasing from 14 in 2001 to 7 in 2019. Its ranking among the BRI countries also dropped from first to seventh position, while its betweenness centrality fell from first to ninth. Thailand’s declining trade status can be explained by the influence of the rice pledge policy under the Yingluck government, which sought to take advantage of Thailand’s monopoly in rice exports to raise the export price and earn more revenue. However, Thailand’s place in the international rice market was quickly occupied by other major cereal trading countries. As a result, it lost many export markets and its central position in the cereal trade. Similarly, Vietnam’s degree centrality fluctuated, dropping from the seventh position among the BRI countries in 2001 to fifth in 2019 as its trade influence gradually declined. Second, Turkey’s trade centrality shifted over the period, with a significant increase in its trading status after 2013. Its degree centrality ranking decreased from four in 2001 to two in 2008 before rapidly increasing after 2013 to reaching the seventh-highest ranking in 2019. During this time, Turkey’s betweenness and eigenvector centrality both rose, ranking it third among 65 BRI countries by the end of the period. Therefore, Turkey’s influence and connectivity within the trade network significantly increased, and its bridging role grew considerably. Turkey has seen a significant increase in its level of economic development since 2000 due to its government’s “neoliberal” economic policy, and the demand for imported cereal has grown in both scale and diversity. Russia is Turkey’s main source of cereal imports, and the scale of cereal trade between the two countries has increased in recent years. Trade flows between the two countries reached $900 million in 2013, the fourth-highest among the BRI countries. By 2019, they had risen to $1.6 billion, the highest bilateral volume among all 65 countries, underlining Turkey’s position as a trade hub.

### 3.4. Core-Periphery Structures of the BRI Region

**Figure 3:**
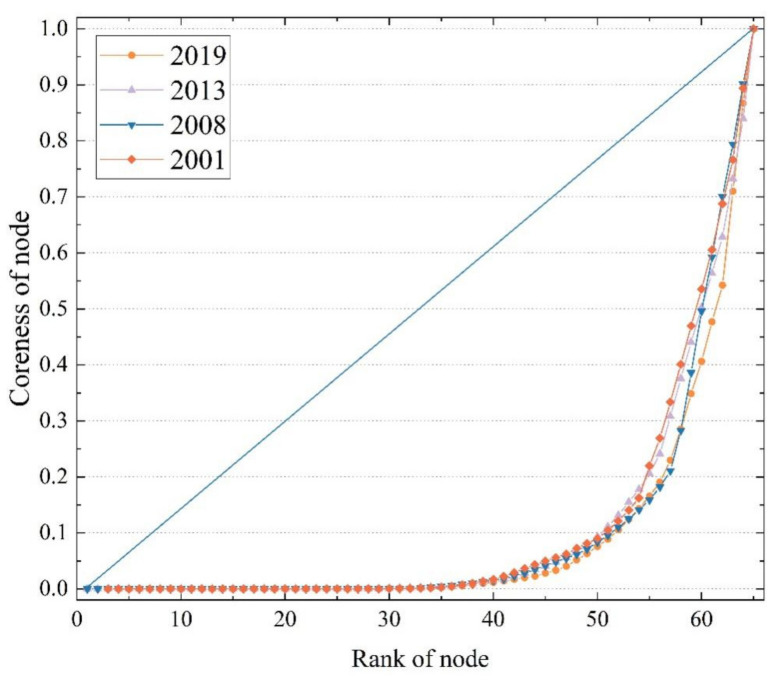
The relationship between coreness and countries’ ranking in the BRI cereal trade networks. Data source: https://comtrade.un.org/data/ (accessed on 14 December 2021), illustrated by the authors.

Based on the full cereal trade networks, we further employed the algorithm of the core-periphery profile to measure the polarization effect of the core-periphery structures in the BRI cereal trade networks. In addition, the coreness of each country reflects the position and role of nodes in the network. Since 2001, the coreness of the cereal trade networks along the BRI has fluctuated from 0.79 to 0.84, indicative of a significant core-periphery structure in which some node countries occupied essential network positions. Over time, the trade agglomeration effect of the core countries within the trade network strengthened considerably. Figure 3 demonstrates a gradual divergence in the nodes’ coreness around the point where their ranks reach 40. The curves are also “J”-shaped, i.e., the nodes with a high rank have extremely high coreness and small numbers, while the nodes with a low rank generally have low coreness and large numbers. The top part of the “J” curve tends to expand outward over time, indicating a decrease in the coreness of some nodes in the high order.

**Figure 4:**
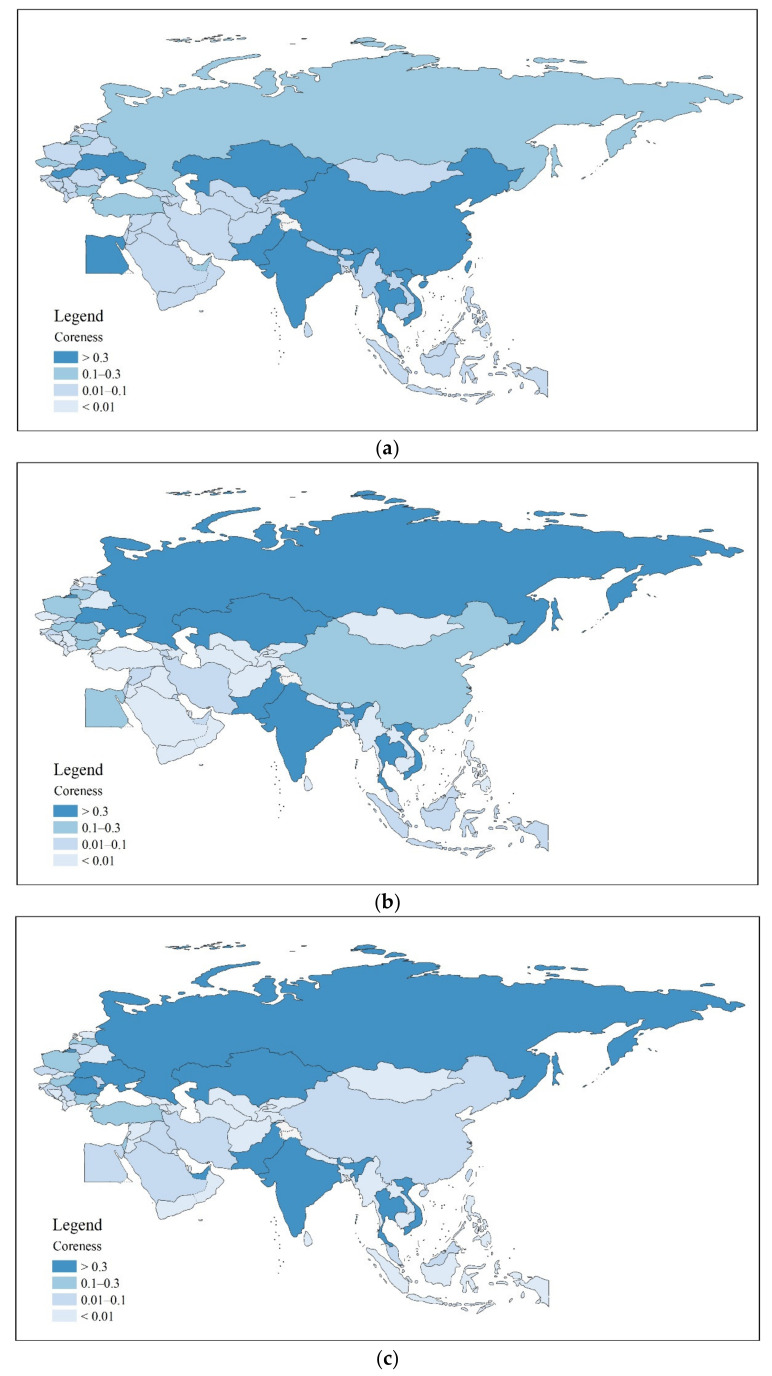

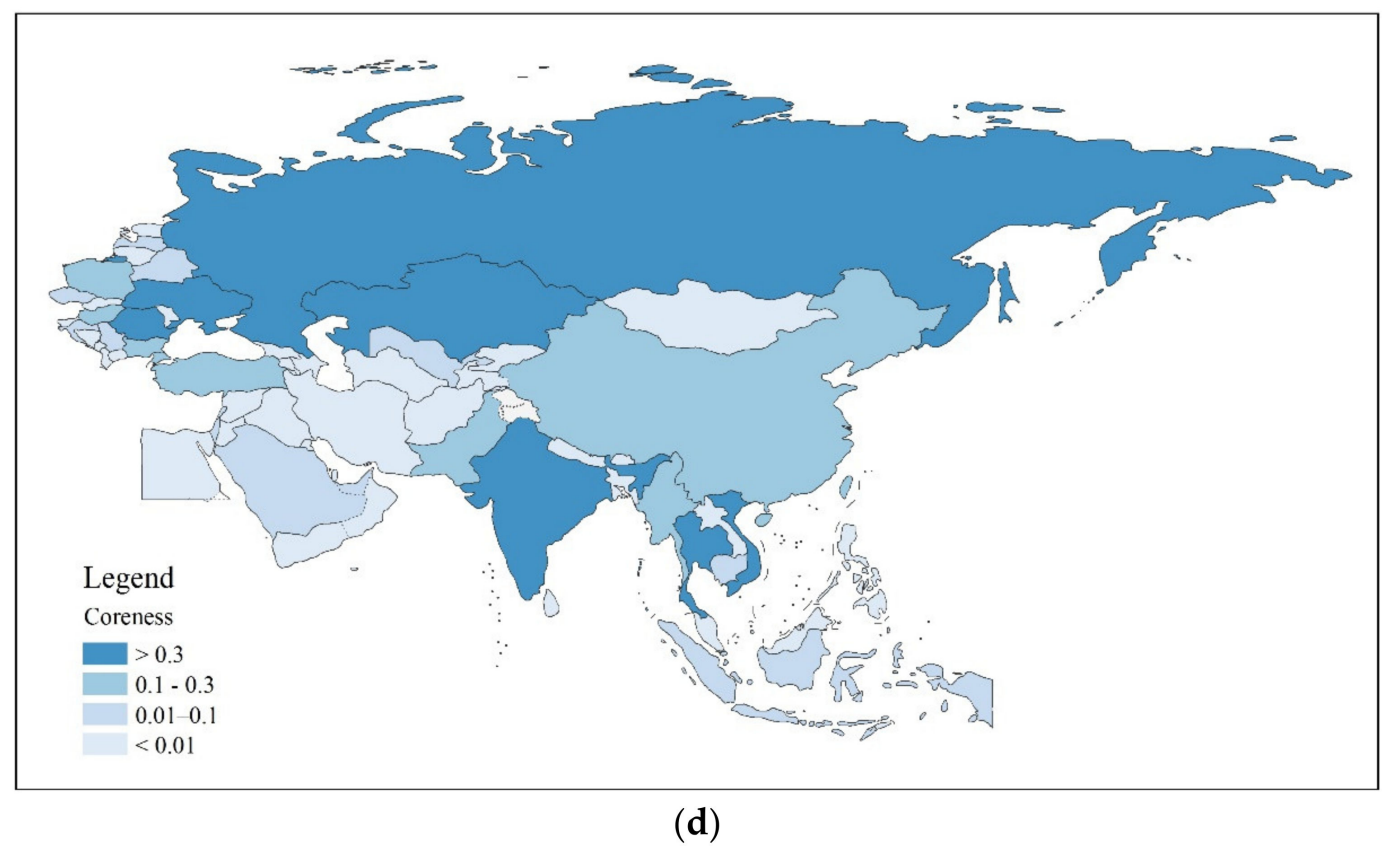
The core-periphery structures of the BRI cereal trade networks (**a**) in 2001, (**b**) in 2008, (**c**) in 2013, and (**d**) in 2019. Data source: https://comtrade.un.org/data/ (accessed on 14 December 2021), illustrated by the authors.

To further analyze the core-periphery structures of the BRI countries in the cereal trade networks, we classify the position of countries in the network into four levels based on their coreness. Countries with a coreness greater than 0.3 are classified as the core structure, countries with a coreness between 0.1 and 0.3 are classified as sub-core structure, countries with a coreness between 0.01 and 0.1 are classified as sub-periphery structures, and countries with coreness less than 0.01 are classified as periphery structure (Figure 4).

Based on these results, there are five points we wish to highlight. First, the number of nodes in the different hierarchical structures changed little over the period. The number of nodes in the core structure decreased from 9 in 2001 to 7 in 2019, while the number of countries in the periphery structure increased slightly from 37 to 39. Second, Ukraine, Kazakhstan, India, Thailand, and Vietnam were permanently part of the cereal trade network’s core structure and export core. Ukraine is a major global cereal exporter, with its extensive black soil, flat and open terrain, and well-developed commercial cereal agriculture making it the world’s leading exporter of wheat. Kazakhstan’s agricultural sector centers on cereal production, and the country also ranks among the world’s top 10 wheat exporters. In contrast, India, Thailand, and Vietnam are the world’s leading rice exporters and control more relationships and resources in the trade network, maintaining their connectedness. Third, Russia’s trade position strengthened to make it the absolute core of the trade network, with its coreness climbing from 0.27 in 2001 to 1 in 2019, bringing it from tenth to first place in the rankings. After Russia joined the World Trade Organization at the end of 2011, the scale of its cereal trade increased, and it became the world’s largest wheat exporter. Fourth, China’s trade position shows a weakening trend and a falling dependence on imports. While China has traditionally been a cereal importing country, its self-sufficiency in rice and wheat has grown in recent years: Cereals are imported to transfer surplus and enrich domestic cereal consumption options. Finally, most cereal import-dominated countries were within sub-periphery structures, with Saudi Arabia, UAE, Egypt, Iran, and Indonesia the leading cereal importers along the BRI. Due to their spatial concentration and large trade volume with the major cereal trading countries, these countries enjoyed fewer and less diverse links with other countries, and thus occupied peripheral positions within the trade network.

## 4. Discussion

### 4.1. Understanding of Cereal Trade Network Structures

Network structures are characterized by complex systems with highly diverse connections, structural complexity, and dynamic evolution. Adopting appropriate methods to clarify the network, extract the backbone structure, and identify the inner order allow the network structure to be fully understood and optimized, improving its performance and enhancing its resilience. To counter the masking of comprehensive information on the network structure by the minor weighted connections, this study applied the concept of top network structure. We extracted the top2 structures of the cereal trade network of the BRI countries, identifying the main trade network of each country’s two most important trading partners. The top network approach clearly and intuitively reveals the structural characteristics of the main trade connections, the factors involved at each level of nodes, and their spatiotemporal dynamics. We found that the BRI facilitated the interdependence development of regional l cereal trade networks, with Russia, India, and Ukraine at their cores, while China’s trade position weakened and its external dependence decreased. These results effectively validate Chen’s findings of a complete non-directional trade network based on the BRI, whether from a global, local or individual country network dynamics perspective [32]. Furthermore, our top network analysis indicated that the main trading networks of some low-volume countries evolved dynamically. To the present time, BRI nations do not have fixed trading partners, and market competitiveness is strong. This finding [32] occupies an unfilled niche in earlier trade network studies, which have previously overlooked relations between countries with smaller trade volumes. Overall, the top network structure demonstrates that the most trading value is produced through trade links with smaller national partners. As an approach, top network identification offers a comprehensive means of capturing the connectivity, high weights, and complexity of cereal trade networks.

Similar to the findings of global cereal trade networks [21], the cereal trade networks of the BRI countries have a significant core-periphery structure. However, there are some differences between the core countries. The core countries of the BRI cereal trade network are Russia, India, and Ukraine, but these countries are not prominent in the studies of global cereal trade networks and are replaced by the United States, the United Kingdom, France, and Canada. This reflects the spatial scale dependence effect of international trade relations [54,55], i.e., that the influence of different levels of network cores is differentiated at the global and regional scales. The characteristics of geographical proximity [56] in the cereal trade are confirmed by this paper. The regional cooperation and degree of regional integration profoundly affect international trade relations, especially in cereal trade [21]. Food security requires a global vision, but it is also crucial to attend to the regional cooperation. By identifying the regional industrial chain and the core forces influencing the regional trade, it is possible to construct a more precise control system that minimizes risk and guarantees the resilience of food security.

### 4.2. Implications for Promoting the BRI Cereal Security

Economic globalization has enabled the international cereal trade and agricultural cooperation to develop. This has guaranteed the global food supply, met the diverse nutritional needs of populations, and promoted national food security [57,58,59,60]. However, the distribution of power in food trade networks and the stability and resilience of supply chains profoundly affect the coherent relationship between trade flows and food security. Significant power asymmetries exist in the actual development of countries due to differences in food supply and demand differences [61]. Food-exporting countries occupy the central positions in trade networks by participating autonomously in trade transactions and controlling more relationships and resources, while import-dependent countries assume more passive positions due to their constant need to ensure the security of their food supply. Therefore, the presence of fewer and more polarized core countries in the food trade network leads to a greater concentration of resources in the trade network, more asymmetrical power relations, and weaker network resilience [62]. However, our study of the BRI cereal trade networks has found that the trade cores have evolved from a relatively diversified to a polarized state. A few core cereal-exporting countries dominate these networks, such as Russia, India, and Ukraine, while the sub-periphery is occupied by cereal-importing countries. This finding aligns with the earlier research into global food trade networks carried out by Wang [9], who found that asymmetrical power relations in trade networks were significant, both globally and among BRI countries. In this type of trade network structure, any instability that affects food production or exports within the core countries, such as natural disasters, political unrest, major public health events, or even the outbreak of war, may affect the food supply security of the entire networks [30,63].

The UN’s Sustainable Development Goals (SDGs) emphasize the need to eliminate hunger and achieve food security [63,64,65]. The global recession triggered by the COVID-19 pandemic at the end of 2019 has caused the international cereal trade to contract, and prices are already at historically high levels. The current war between Russia and Ukraine involves two of the world’s leading cereal producers and exporters. War profoundly affects the production and export of cereal, causing large short-term price fluctuations, affecting the long-term organization of the industry, and spreading the crisis to additional countries and populations through trade. From the network perspective, the Russia-Ukraine war has disturbed the stability of the two core node countries and will disrupt or even rupture the local food industry and supply chain. Furthermore, the current war will severely threaten the food security of countries that are highly dependent on food imports and increase the vulnerability of the entire trade network, further worsening regional food insecurity.

Food security is a fundamental issue for the survival of humankind, and the destiny of all countries is closely intertwined. Ensuring global and regional food security is fully aligned with the development concept of a community of human destiny advocated by the BRI. Cereal trade networks play a crucial role in ensuring the resilience of trade among the BRI countries and should be promoted as a model of global cereal trade partnership in achieving food security. With that in mind, the following policy recommendations are made to strengthen the cereal trade within the BRI. First, the trade network structure should be optimized, and the diversification of the cereal trade should be promoted. More countries should be encouraged to participate fully in the BRI cereal trade networks, thus increasing the abundance of the network system. Countries with high cereal import dependence or concentrations should seek to expand the scope of their trade partnerships and moderately diversify their sources of cereal supply. Second, the capacity of countries to increase food production should be enhanced, and food reserve capacity should be strengthened. This entails a gradual change from promoting cereal production to supporting the construction of the whole cereal supply chain in multiple directions in order to build a comprehensive supply chain system. Third, the concept of inclusive cooperation should be upheld, and a regional food security community should be built. The BRI countries are advised to implement the concept of inclusive globalization actively, promote policies and measures to facilitate smooth trade flows, and call for the establishment of multilateral commitments among countries to refrain from imposing restrictions on the export of agricultural raw materials, such as food and fertilizers. They should also improve the structural connectivity of regional cereal trade networks, strengthen special cooperation mechanisms between countries in food-related fields, and build a close-knit community of destiny. Finally, risk prediction and assessment mechanisms should be established to improve the risk resistance of trade networks. The academic community should conduct additional studies on the cereal trade networks of countries in the BRI region. The cereal production and circulation systems and regional political, economic, and security situations should also be closely monitored to prevent and mitigate the impact of unexpected situations on the trade network system.

## 5. Conclusions

The BRI plays an essential and positive role in promoting regional trade integration. Since ancient times, agricultural cooperation and trade have been key to trade cooperation among countries along the Silk Road. Based on the cereal trade networks database, we have examined the structural evolution of cereal trade networks in the BRI region using a systematic network research approach and measured changes in the relative position of different countries between 2001 and 2019.

Our findings suggest that the cereal trade of the BRI countries continued to grow throughout the period, from $3.809 billion in 2001 to $28.508 billion in 2019. As a result, the cereal trade in the BRI region formed closely linked and complex trade networks. The BRI significantly boosted cereal trade in participating countries, deepening the interdependence of cereal trade between these countries and strengthening their backbone connections. Geographical proximity is significant to the backbone network structure of cereal trade along the BRI. India, Russia, and Ukraine were the most important cereal trading partners and the absolute centers of the cereal trade network along the BRI, influencing the entire cereal trade network structure. During the period, the main trade partners of certain countries shifted, indicating that fixed international cereal trading partnerships had not yet been established, and competition among suppliers was apparent.

Cereal trade networks along the BRI possess significant core-periphery structures, and the trade agglomeration effect of the core countries has increased over time. Since 2001, the core has developed from relatively diversified to polarized, with significant power asymmetries among countries due to the relationship between cereal supply and demand. Major cereal exporters have long been at the core, controlling more relationships and resources in the trade network, while cereal import-dominated countries generally occupy a less significant trading position within the sub-periphery structures.

Since the COVID-19 pandemic at the end of 2019 severely disrupted the international food trade system and continues at the time of writing, any subsequent changes are outside the scope of this study. Therefore, we only conducted cereal trade studies up to 2020 and did not explore the time period of 2020 and beyond. Moreover, the ongoing Russia-Ukraine war will profoundly impact the international cereal trade system. Current cereal trade patterns within and beyond the BRI are likely to undergo dynamic adjustments and reconstruction. In future research, we will conduct studies to assess the impacts of the crisis, such as the COVID-19 pandemic and the Russia-Ukraine war on global and regional food security and develop the corresponding strategies for risk prevention and control, crisis management, and resilience assessment of the BRI cereal trade networks in different contexts. 

## Figures and Tables

**Table 1:** The scope of the BRI in this study.

Region	Countries
China, Mongolia, and Russia	China, Mongolia, Russia
11 Countries in Southeast Asia	Indonesia, Thailand, Malaysia, Vietnam, Singapore, Philippines, Myanmar, Cambodia, Laos, Brunei, Timor-Leste
8 Countries in South Asia	India, Pakistan, Bangladesh, Sri Lanka, Afghanistan, Nepal, Maldives, Bhutan
5 Countries in Central Asia	Kazakhstan, Uzbekistan, Turkmenistan, Kyrgyzstan, Tajikistan
19 Countries in West Asia and the Middle East	Turkey, Iran, Syria, Iraq, UAE, Saudi Arabia, Qatar, Bahrain, Kuwait, Lebanon, Oman, Yemen, Jordan, Israel, Palestine, Armenia, Georgia, Azerbaijan, Egypt
19 Countries in Central and Eastern Europe	Poland, Czech Republic, Slovakia, Hungary, Slovenia, Croatia, Romania, Bulgaria, Serbia, Montenegro, Macedonia, Bosnia and Herzegovina, Albania, Estonia, Lithuania, Latvia, Ukraine, Belarus, Moldova

**Table 2:** The centrality indicators of international cereal trade of the BRI countries.

**2001**						**2008**					
**Country**	**DC**	**Country**	**BC**	**Country**	**E.C.**	**Country**	**DC**	**Country**	**BC**	**Country**	**E.C.**
Thailand	14	Thailand	542.47	Thailand	1.00	Russia	14	India	527.27	Russia	1.00
India	10	Hungary	380.51	China	0.49	Thailand	13	Russia	515.36	Kazakhstan	0.80
Hungary	9	India	363.35	India	0.45	India	12	Thailand	498.61	India	0.52
Russia	9	Russia	343.42	Malaysia	0.39	Kazakhstan	12	Hungary	473.33	Ukraine	0.41
Kazakhstan	8	Saudi Arabia	321.44	Indonesia	0.36	Hungary	9	Pakistan	398.97	Bangladesh	0.38
Vietnam	7	Romania	298.70	Vietnam	0.35	Pakistan	9	Kazakhstan	288.89	Lebanon	0.38
China	6	Kazakhstan	198.01	Iran	0.35	Ukraine	9	Ukraine	229.22	Mongolia	0.38
Pakistan	6	Vietnam	191.31	Iraq	0.35	Vietnam	5	Albania	220.47	Azerbaijan	0.38
Ukraine	6	China	190.53	Singapore	0.33	Bangladesh	4	Romania	190.71	Georgia	0.38
Belarus	5	Iran	188.17	Bahrain	0.31	China	4	Oman	161.87	Turkmenistan	0.38
**2013**						**2019**					
**Country**	**DC**	**Country**	**BC**	**Country**	**EC**	**Country**	**DC**	**Country**	**BC**	**Country**	**EC**
India	20	India	1013.46	India	1.00	Russia	19	Russia	653.02	Russia	1.00
Russia	15	Saudi Arabia	674.09	Russia	0.44	Ukraine	14	Ukraine	515.32	Ukraine	0.80
Ukraine	10	Russia	628.80	Pakistan	0.42	India	13	Turkey	373.41	Turkey	0.46
Pakistan	8	Ukraine	389.05	Saudi Arabia	0.42	China	8	Romania	360.77	India	0.41
Saudi Arabia	8	Hungary	227.62	Iran	0.40	Kazakhstan	8	Hungary	338.99	Egypt	0.37
Thailand	8	Vietnam	165.66	Vietnam	0.38	Pakistan	8	Pakistan	300.92	Indonesia	0.35
Vietnam	7	Romania	163.65	United Arab Emirates	0.33	Thailand	7	India	291.16	Bangladesh	0.35
China	6	Serbia	159.37	Ukraine	0.31	Turkey	7	China	285.31	Israel	0.35
Iran	6	Iran	141.47	Malaysia	0.28	Hungary	6	Thailand	186.11	China	0.33
Kazakhstan	6	China	138.98	Yemen	0.26	Romania	6	Kazakhstan	129.92	Armenia	0.33

Data source: https://comtrade.un.org/data/ (accessed on 14 December 2021).

## Data Availability

The new data created in this study are available on request.
